# Supplementary material for: Unexpected polymerization mechanism of dilignol in the lignin growing
Source: R Soc Open Sci. 2019 Jul 24;6(7):190445. doi: 10.1098/rsos.190445 (PMC6689591; doi:10.1098/rsos.190445)
Supplement: Supplementary information from Unexpectable polymerization mechanism of dilignol in the lignin growing [file rsos190445supp1.pdf]

Electronic Supplementary Materials

Unexpected polymerization mechanism of dilignol in the lignin growing

Yasuyuki Matsushita,*[a] Yuto Oyabu [a] Dan Aoki [a] and Kazuhiko Fukushima [a]

[a] Graduate School of Bioagricultural Sciences, Nagoya University, Furo-cho, Chikusa-ku, Nagoya, Aichi, 464-8601, Japan. E-mail: ysmatsu@agr.nagoya-u.ac.jp

Table of Contents

Fig. S1 NMR spectra of **2** P2
Fig. S2 NMR spectra of **3** P3
Fig. S3 NMR spectra of **4** P4
Fig. S4 NMR spectra of **5** P5
Fig. S5 NMR spectra of **6** P6
Fig. S6 NMR spectra of **8** P7
Fig. S7 NMR spectra of β-5 dilignol **II**. P8
Fig. S8 NMR spectra of ^{13}C labeled β-5 dilignol **^{13}C-II**. P9

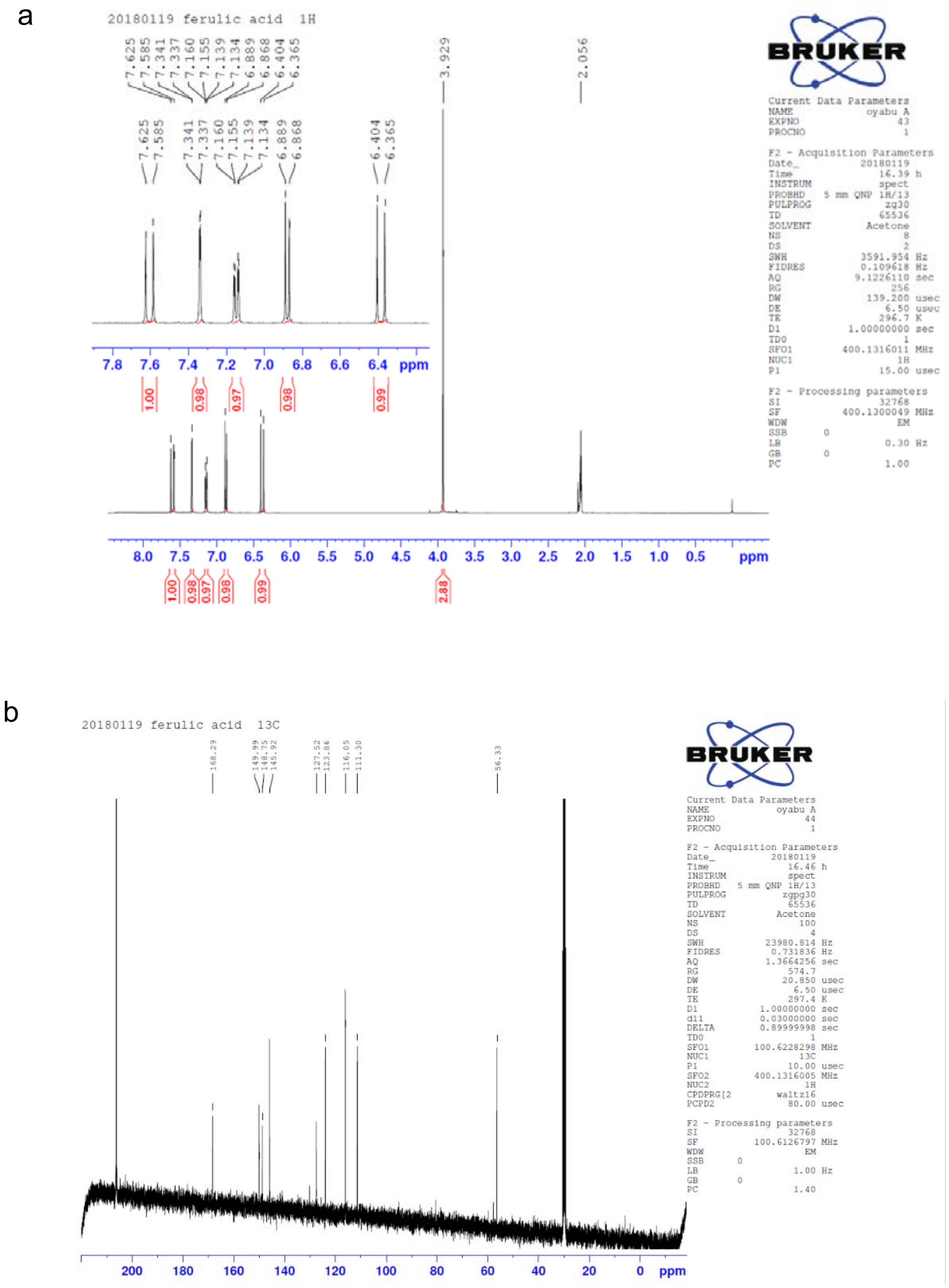

Fig. S1 NMR spectra of **2**. a, ^{1}H NMR spectrum, b, ^{13}C NMR spectrum.

a

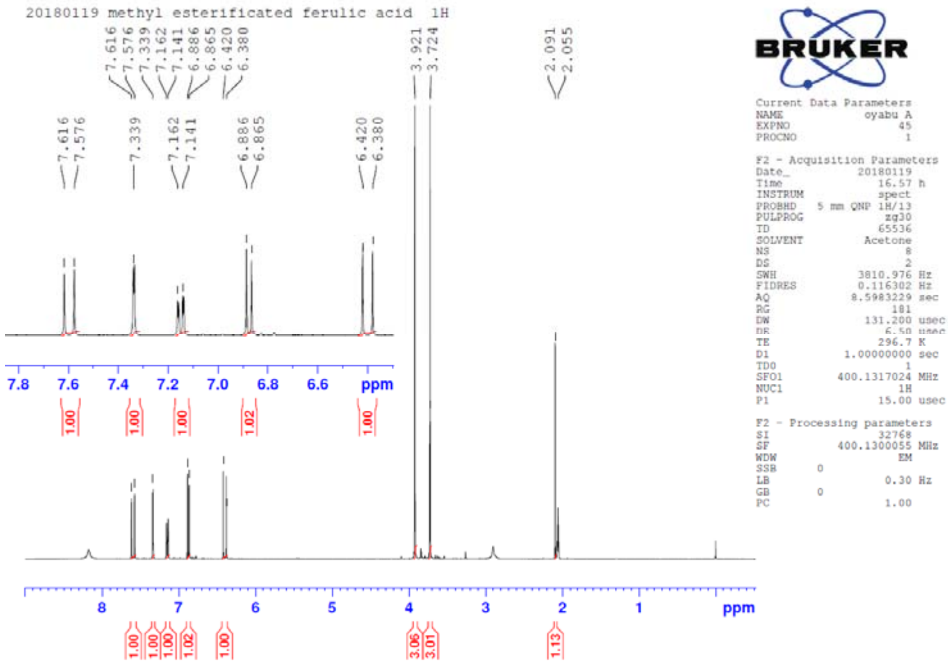

b

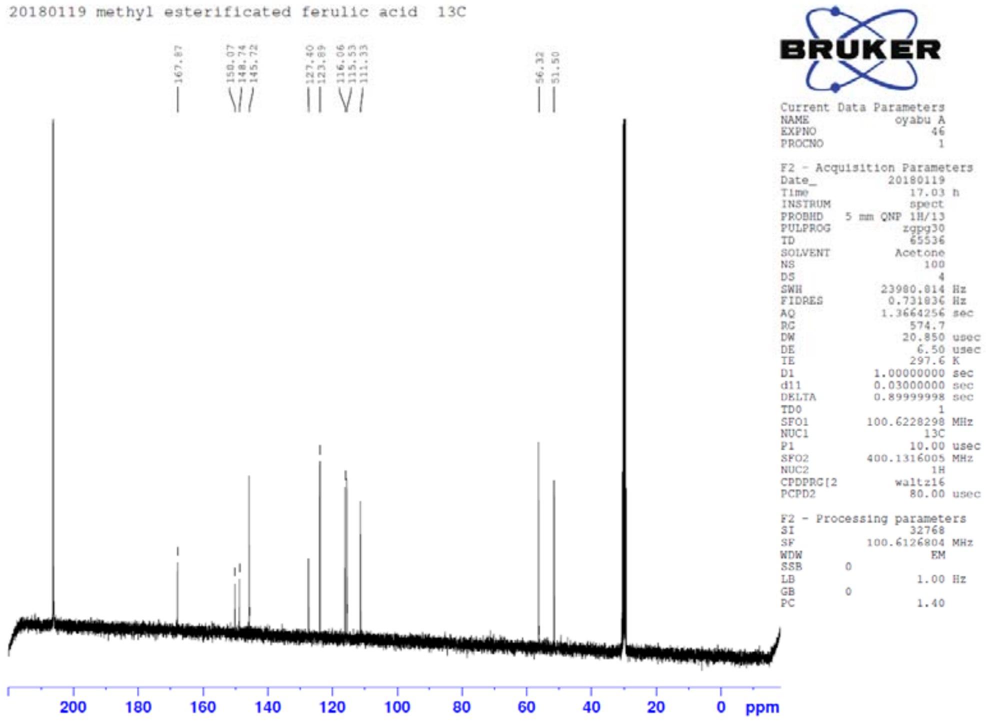

Fig. S2 NMR spectra of **3**. a, ^{1}H NMR spectrum, b, ^{13}C NMR spectrum.

a

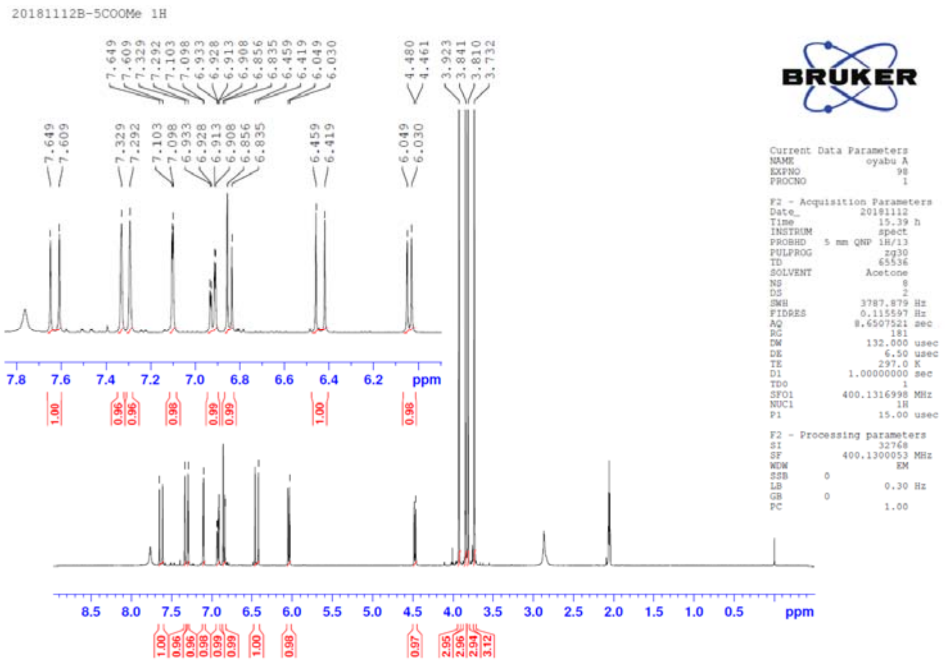

b

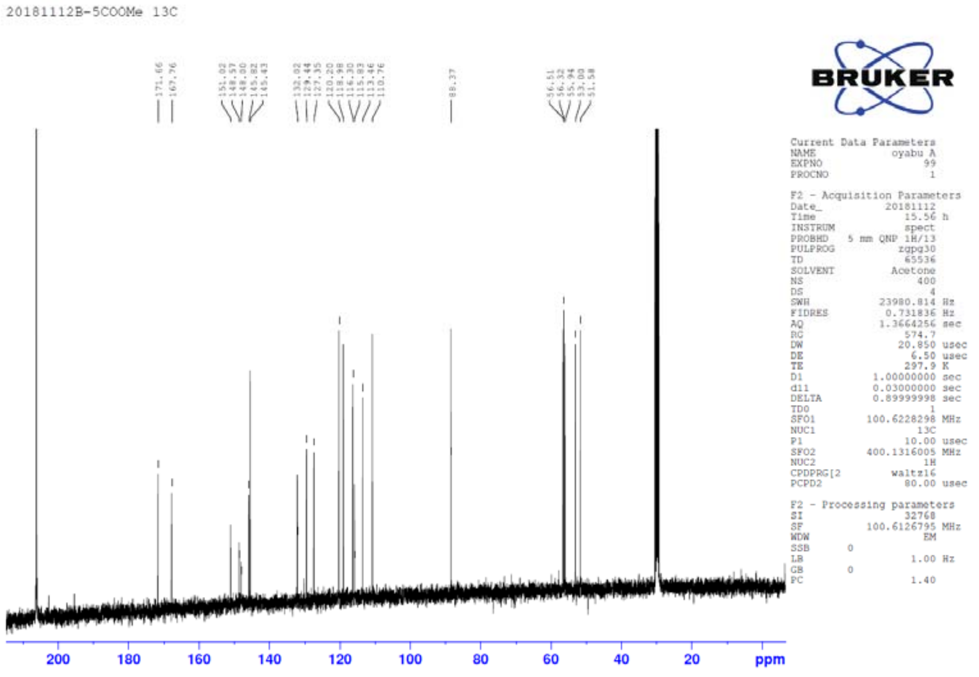

Fig. S3 NMR spectra of **4**. a, ^{1}H NMR spectrum, b, ^{13}C NMR spectrum.

a

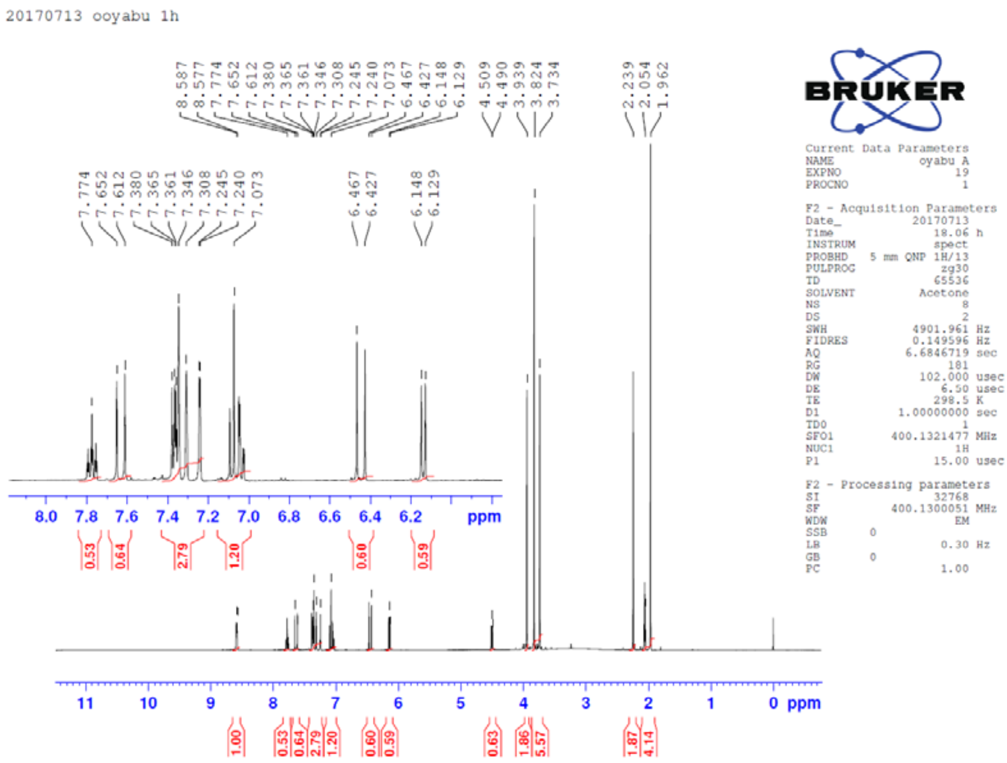

b

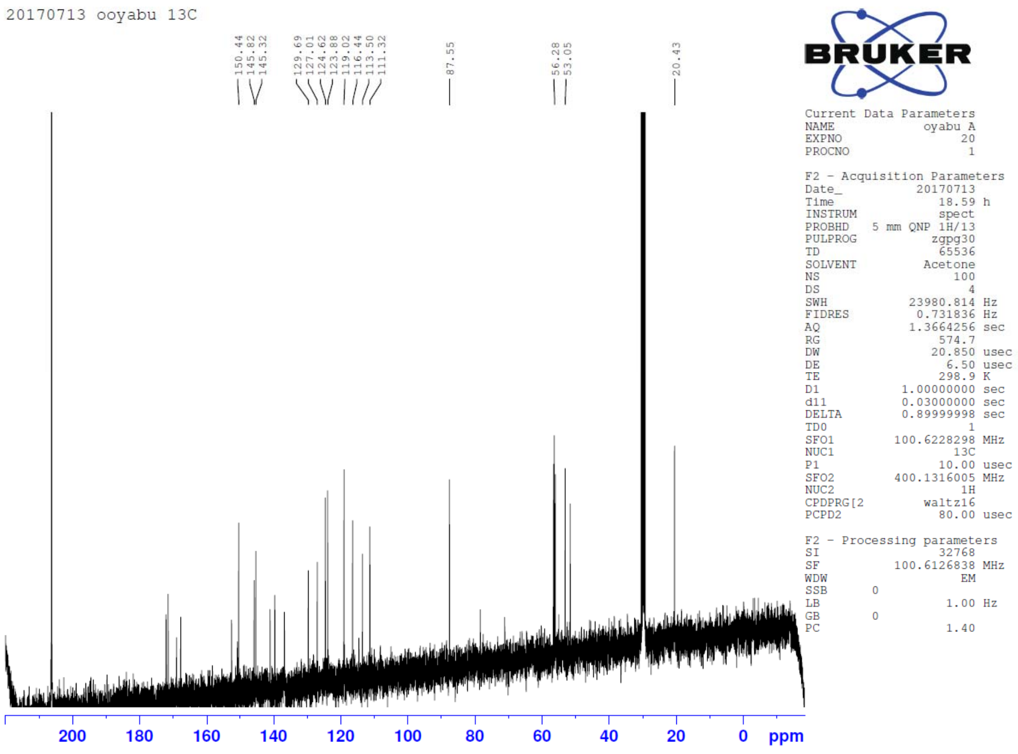

Fig. S4 NMR spectra of **5**. a, ^{1}H NMR spectrum, b, ^{13}C NMR spectrum.

a

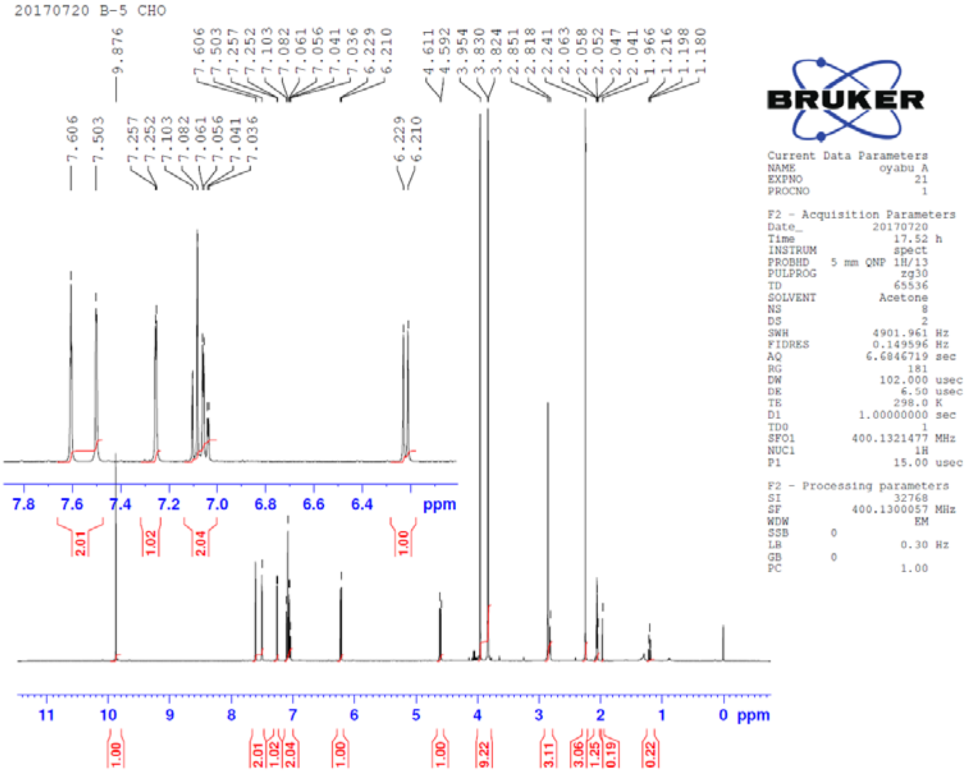

b

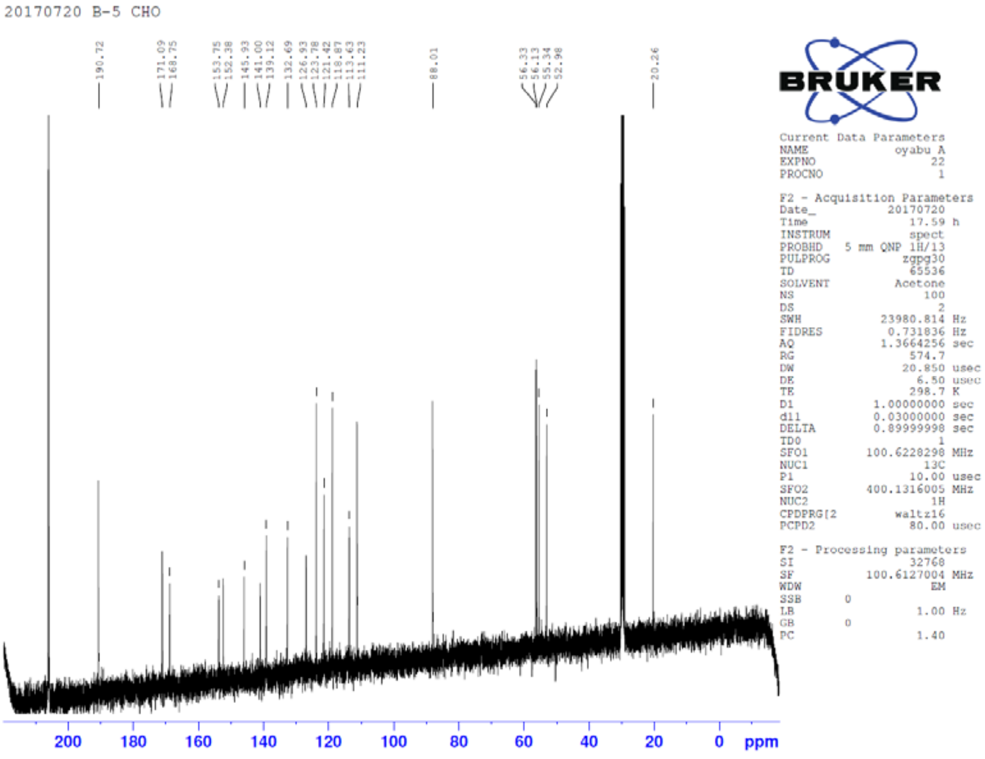

Fig. S5 NMR spectra of **6**. a, ^{1}H NMR spectrum, b, ^{13}C NMR spectrum.

a

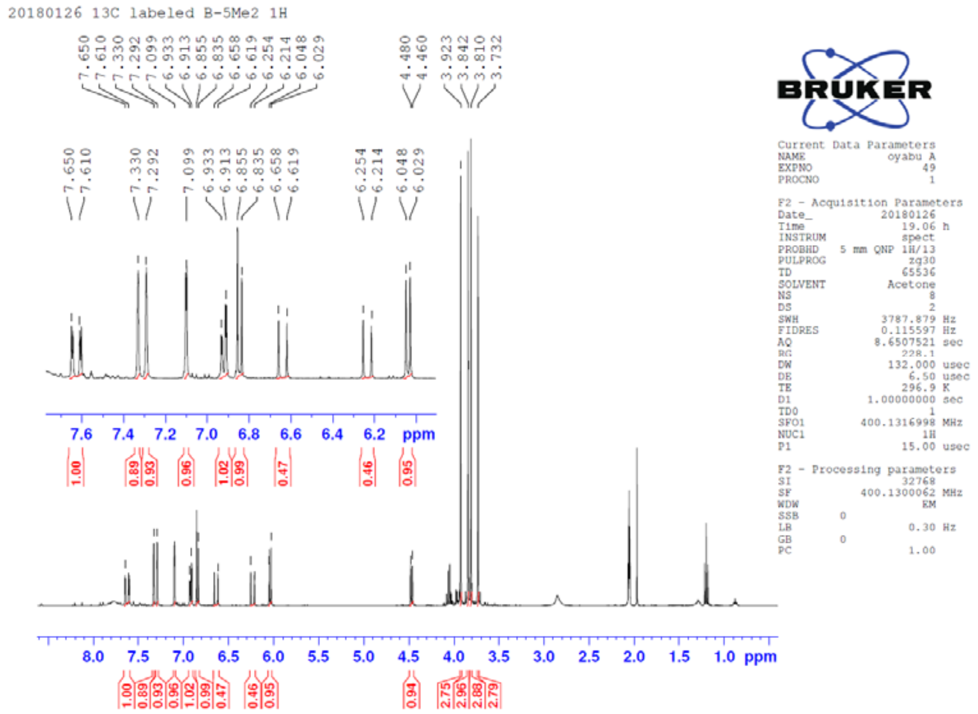

b

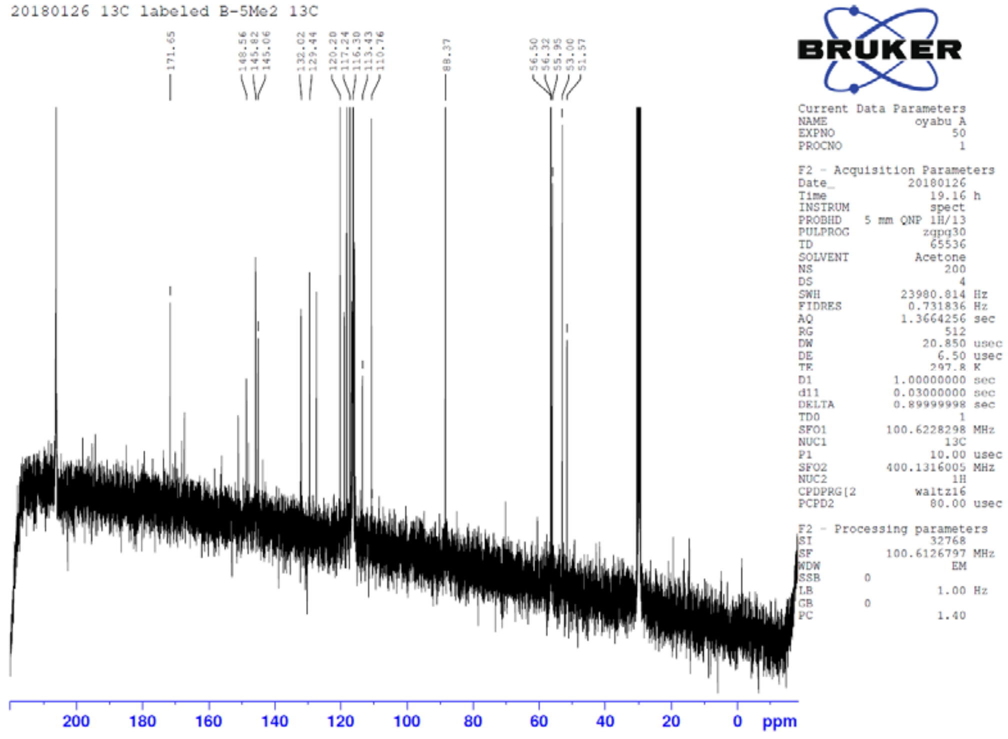

Fig. S6 NMR spectra of **8**. a, ^{1}H NMR spectrum, b, ^{13}C NMR spectrum.

a

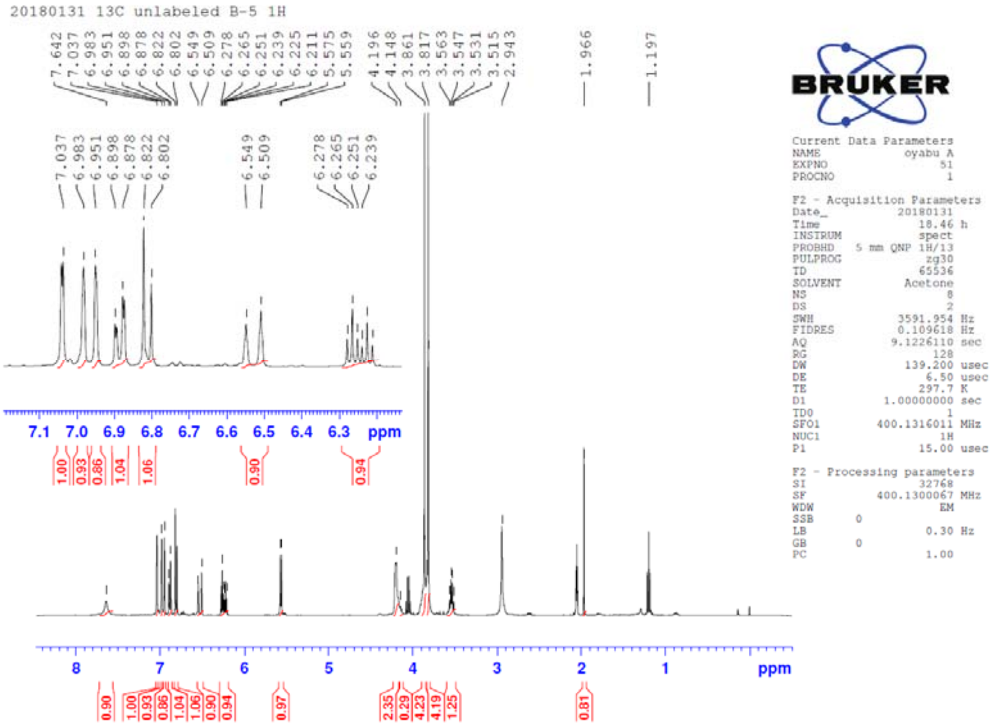

b

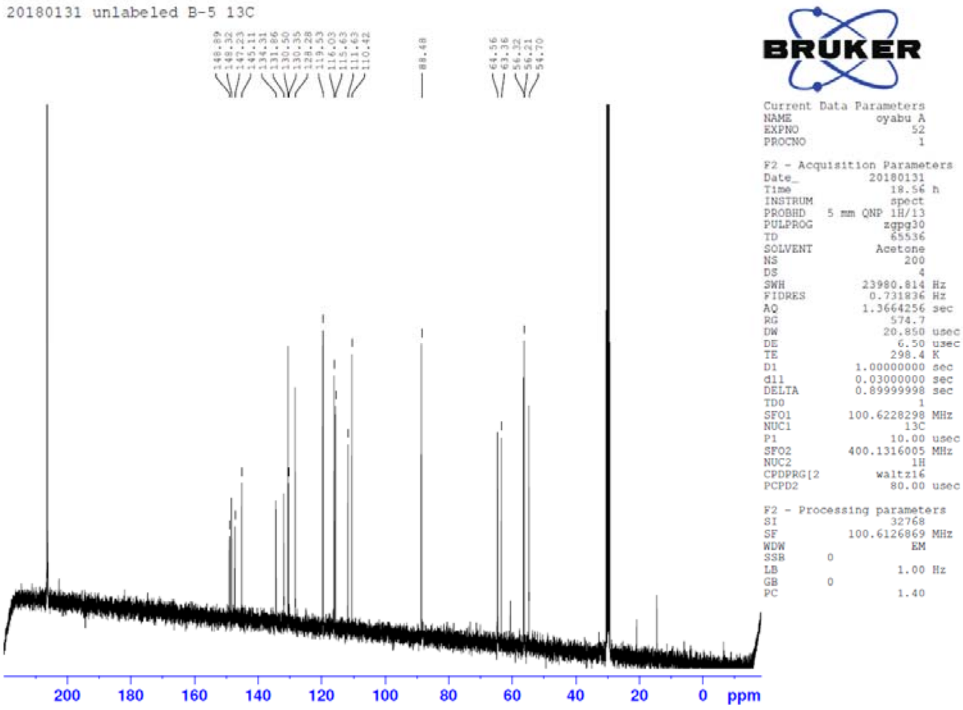

Fig. S7 NMR spectra of β-5 dilignol **II**. a, ^{1}H NMR spectrum, b, ^{13}C NMR spectrum.

a

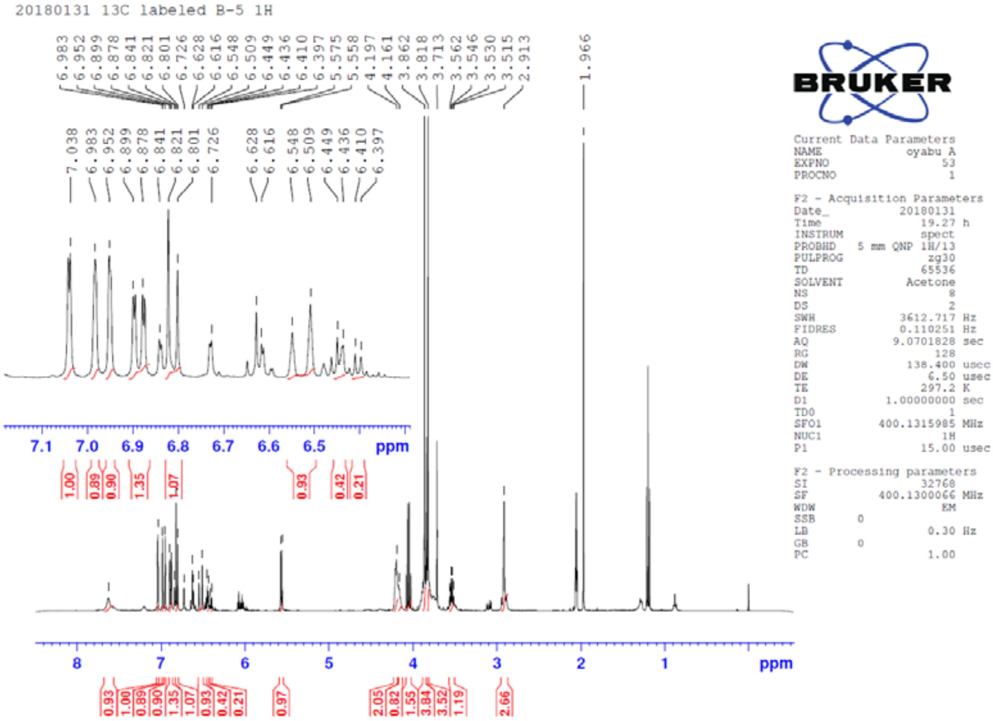

b

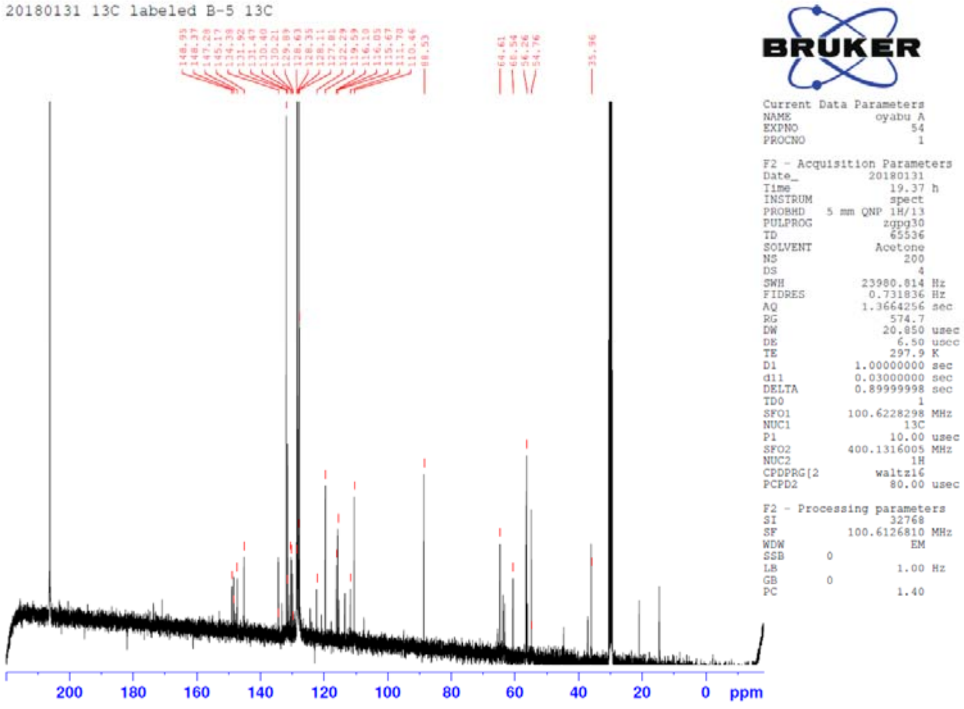

Fig. S8 NMR spectra of ^{13}C labeled β-5 dilignol **^{13}C-II**. a, ^{1}H NMR spectrum, b, ^{13}C NMR spectrum.
